# Pooling data for primary total knee implants across national registries: is the same implant used in multiple registries and for the same patient group? An observational study

**DOI:** 10.2340/17453674.2025.43476

**Published:** 2025-04-17

**Authors:** Lotje A HOOGERVORST, Rob G H H NELISSEN, Liza VAN STEENBERGEN, Alma B PEDERSEN, Eskild Bendix KRISTIANSEN, Martin LINDBERG-LARSEN, Marina TORRE, Enrico CIMINELLO, Riccardo VALENTINI, Alexander W GRIMBERG, Yinan WU, Perla J MARANG-VAN DE MHEEN

**Affiliations:** 1 Department of Orthopaedics, Leiden University Medical Center, Leiden, the Netherlands; 2 Dutch Arthroplasty Register (LROI), ‘s- Hertogenbosch, the Netherlands; 3 Department of Clinical Epidemiology, Aarhus University Hospital, Aarhus, Denmark; 4 Department of Clinical Medicine, Aarhus University, Aarhus, Denmark; 5 The Danish Knee Arthroplasty Register; 6 Department of Orthopedic Surgery and Traumatology, Odense University Hospital, Odense, Denmark; 7 Italian Implantable Prostheses Registry, Italian National Institute of Health, Rome, Italy; 8 German Arthroplasty Registry – EPRD, Berlin, Germany; 9 Safety & Security Science and Centre for Safety in Healthcare, Delft University of Technology, Delft, the Netherlands

## Abstract

**Background and purpose:**

Pooling data on the performance of total knee (TK) implants across registries is only possible if the same TK implant is used across multiple registries and if used in patients with similar characteristics. We assessed to what extent specific TK implants: (i) are used across multiple registries or only in a single registry; and (ii) differ in patient characteristics between registries.

**Methods:**

All primary TK implants implanted between January 2020 and December 2021 in the Danish, Dutch, German, and Italian registries were included. We determined the number of registries using a specific TK implant (based on combined femoral-tibial component brand name and fixation/congruency/mobile bearing insert/patella usage). Patient characteristics (age/body mass index [BMI]/sex/diagnosis osteoarthritis) were compared across registries for TK implants used in ≥ 2 registries ≥ 100 times.

**Results:**

813 different TK implants (577,351 procedures) were used across the 4 registries, of which 53 TK implants (7%) were used in 1 registry (8,000 procedures). 760 different TK implants (569,351 procedures; 99%) were used in ≥ 2 registries of which 47 different TK implants (393,954 procedures; 68%) were used in ≥ 2 registries and ≥ 100 times. Statistically and clinically significant differences in age for the same TK implant across registries were observed for 29 TK implants (62%) and 3 TK implants (6%), respectively; for other characteristics these were for BMI 30 (64%) and 0 (0%) TK implants; for male proportion 23 (49%) and 17 (36%) TK implants; and for diagnosis of osteoarthritis 42 (89%) and 34 (72%) TK implants, respectively.

**Conclusion:**

Most specific TK implants and TK procedures were used across multiple registries, but they were often used in patients with different characteristics. This has an impact on comparing implant performances between registries.

Arthroplasty registries are well suited to assess the safety and performance of total knee (TK) implants, as most registries publish annual reports including survivorship data of specific TK implants [1-3]. Many registries have outlier procedures in place to detect implants with significantly higher revision [1,2,4]. Several factors may influence TK-implant performances, including implant-related factors such as implant materials or the production process, for which the Optetrak case showed that implant-related factors resulted in significantly worse performance [5-7].

Patient characteristics (e.g., age, sex, and body mass index [BMI]) can also affect the performance of TK implants [8-10]. To compare performance of a specific a TK implant across registries, characterized by brand name and implant characteristics to avoid camouflage, it is thus important to consider the characteristics of patients receiving that specific TK implant. Few studies have assessed differences in patient characteristics across countries [11-13], with most studies only focusing on variations in preoperative pain and function. Importantly, all studies analyzed the entire group of TK implants (e.g., all cemented TK implants) rather than analyzing differences for specific TK implants (characterized by a specific brand and implant characteristics like fixation and congruency). Hence, a more comprehensive analysis of the similarities and differences in patients receiving a specific TK implant is required to better understand possible differences in safety and performance of TK implants across registries. Such a comprehensive analysis is also needed to pool data across countries/registries or when performing distributed meta-analyses, where ensuring the same patient mix is crucial for fair comparison of safety and performance.

The aim of this study was to assess, across national registries, to what extent specific TK implants: (i) are used across multiple registries or only in a single registry; and (ii) differ in patient characteristics between registries.

## Methods

### Design and setting

The study was designed as a comparative observational study including data from 4 national European arthroplasty registries. 8 European national registries were asked to participate. Although all registries showed interest in doing so, this required some effort to make the standard script applicable to the registry, conduct the analyses, and send the data, which the following 4 European registries managed to do: the Danish Knee Arthroplasty Register (DKR), the Dutch Arthroplasty Register (LROI), the German Arthroplasty Register (EPRD), and the Italian Arthroplasty Registry (RIAP). Regarding the EPRD, only registry data with complete linkage to insurance data was included (10.5% of all TK procedures) [14]. TK implant-level completeness for the included 4 registries ranged from 59% (RIAP) to 97% (LROI) [15,16]. Aggregated TK-implant-level data was retrieved from each registry, including all patients receiving a primary TK implant between January 1, 2010 and December 31, 2021. In addition, each registry provided the number of unicompartmental knee (UK) implant procedures in this period, as different use of UK implants across registries may have been impacted by patient characteristics [13]. The study is reported according to STROBE guidelines.

### Categorization of TK implants

Groups of comparable TK-implant constructs were defined based on the following implant characteristics: implant–bone fixation (i.e., fixation), tibial insert–femoral congruency (i.e., congruency), mobile bearing insert, and patella usage (Table 1). Within each TK construct, the brand name of both the femoral and tibial component was used to indicate a specific TK implant.

**Table 1:** Implant characteristics used to categorize TK constructs in primary arthroplasty, and their definitions according to the LROI implant library [30]

**Fixation:**	***Cemented; cementless; hybrid*** – LROI definition: femoral component is cementless, tibial and/or patellar components are cemented
**Congruency:**	***Mega prosthesis*** (i.e., maximal-hinged or mega tumor resection prosthesis) – LROI definition of hinged: a component that only allows for flexion and extension through a fixed axis and provides collateral as well as posterior ligament stability
	***Fully congruent*** (high posterior peg of liner) – LROI definition of fully: a component that only allows for flexion and extension through a fixed axis and provides collateral as well as posterior ligament stability
	***Posterior*** (i.e., posterior stabilized) – LROI definition: both cruciate ligaments removed
	***Medial pivot*** – LROI definition: the medial pivot knee design has a highly congruent medial liner–femoral component contact
	***Minimal*** (i.e., minimally congruent): retaining of posterior cruciate ligament (CR) – LROI definition: retaining of medial CR
	***Bicruciate retaining*** – LROI definition: both cruciate ligaments retained
**Bearing insert:**	***Fixed*** (i.e., non-mobile) – LROI definition: component that is not intended to move relative to its interface component
	***Mobile/rotating*** (i.e., a tibial insert is intended to move on its metal tibial component)
	LROI definition of mobile: a component that is intended to move relative to its interface component
	LROI definition of rotating: a component that is intended to move relative to its interface component. Rotating: where the component moves in an inward and outward direction
**Patella usage:**	***No; yes***

### Patient characteristics

For each specific TK implant in a registry, the number of procedures as well as the following patient characteristics were retrieved: (i) mean age (standard deviation [SD]); (ii) mean BMI (SD); (iii) percentage male sex, and (iv) percentage of patients with the diagnosis osteoarthritis. The registers differed in their classification of the initial diagnosis, and we calculated the percentage of patients with the diagnosis osteoarthritis in the following way for each registry:

- DKR: the number of patients with primary osteoarthritis as the initial diagnosis, relative to the total number of patients. Other diagnoses include rheumatoid arthritis, sequelae after tibia/femur condyle fracture, sequelae after patellar fracture, secondary arthrosis after meniscectomy, hemophilia, cancer, or other.
- EPRD: the number of patients with primary osteoarthritis as the initial diagnosis, relative to the total number of patients. Other diagnoses include post-traumatic osteoarthritis, secondary osteoarthritis, or other.
- LROI: the number of patients with osteoarthritis as the initial diagnosis, relative to the total number of patients. Other diagnoses include post-traumatic, rheumatoid arthritis, osteonecrosis, or other.
- RIAP: the number of patients with primary osteoarthritis as the initial diagnosis, relative to the total number of patients. Other diagnoses include post-traumatic osteoarthritis, rheumatoid arthritis, neoplasia, osteonecrosis, or other.

### Statistics

Descriptive statistics were used to assess the number of registries in which each TK construct (based on implant characteristics: fixation, congruency, mobile bearing insert, and patella usage) was used. We also calculated the percentage of UK-implant procedures reported in each registry relative to all knee (i.e., both TK and UK) implant procedures used.

For each specific TK implant used in ≥ 2 registries and used ≥ 100 times in each registry, we compared patient characteristics across registries. The criterion of ≥ 100 TK implants used per registry was added to ensure sufficient sample size for meaningful analysis. First, we calculated for all patients receiving a TK implant across registries: the mean (SD) age and BMI, as well as the percentage of male sex and patients with osteoarthritis. Thereafter, for each registry and specific TK implant, we calculated a confidence interval around the mean or percentage, using the SD and total number of patient procedures. Statistically significant differences were determined by non-overlapping confidence intervals between registries [17]. As statistical significance does not equal clinical relevance, we applied the commonly used threshold of a ≥ 10% difference (i.e., 10% difference on the 0 to 100% percentage scale) to determine a clinically relevant difference for male sex and osteoarthritis diagnosis, and for the continuous variables age and BMI we used thresholds of a ≥ 5 years difference and a ≥ 5 points difference, respectively [18,19]. These commonly used thresholds are determined in a large cohort study (including 4,183 patients) and in a Delphi study (i.e., the assessment of quality in the lower limb Arthroplasty “AQUILA” initiative) including 44 orthopedic experts.

### Ethics, data sharing plan, funding, use of AI, and disclosures

This work was supported by the European Union Horizon 2020 Research and Innovation Program (grant number 965246) and was part of the Coordinating Research and Evidence for Medical Devices (CORE-MD) project. AI tools were not used in our submission. Complete disclosure of interest forms according to ICMJE are available on the article page, doi: 10.2340/17453674.2025.43476

## Results

### Inclusion of TK constructs

Based on the combination of implant characteristics (i.e., 3 fixation types, 6 congruency types, 2 mobile bearing insert types [yes/no], and patella usage [yes/no]) 72 TK constructs would be possible theoretically, of which 9 (13%) were not used in any of the 4 registries or did not exist (Figure). 63 TK constructs (577,351 procedures; 813 different TK implants) were used in the 4 registries. Of these, 25 (40%) TK constructs were used in a single registry, including 53 out of 813 (7%) different TK implants and 8,000 out of 577,351 (1%) procedures. 27 (71%) of the remaining 38 TK constructs (175,397 procedures; 713 different TK implants) did not have specific TK implants used ≥ 100 times in ≥ 2 registries. Thus, 11 TK constructs, considering 47 specific TK implants and 393,954 (68%) procedures, were included in the comparison of patient characteristics between registries (Figure, Table 2). Of note, no specific TK implants with mobile/rotating bearing inserts were used ≥ 100 times in ≥ 2 registries. Overall, 206 TK implants with mobile/rotating bearing inserts were used across registries but 183 of these were used in only 1 registry and 23 TK implants with mobile/rotating bearing inserts were used < 100 times.

**Figure:**
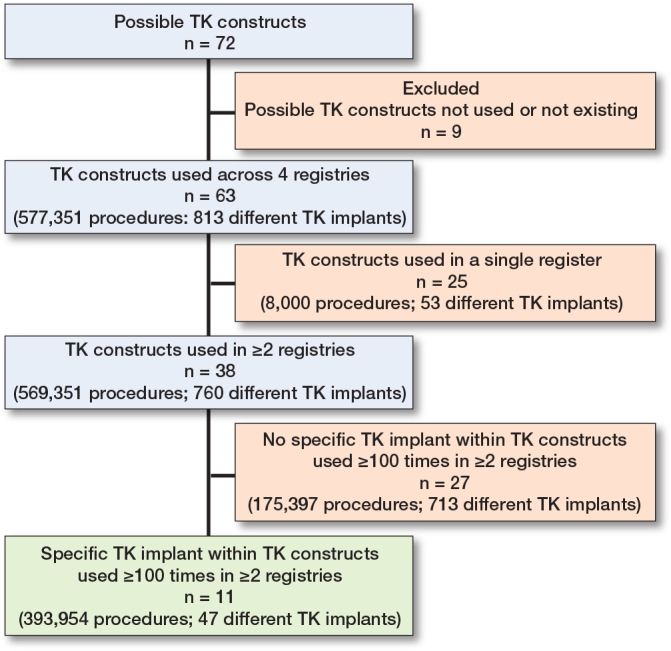
Use of specific TK implants across registries and included TK implants in comparison of patient characteristics.

**Table 2:** TK constructs used ≥ 100 times in ≥ 2 registries

TK construct, see below				Number of implants used in registry					TK implant – Brand name
F	C	M	P	Total	DKR	EPRD	LROI	RIAP	femoral component – tibial component
C	F	F	No	991	–	794	197	–	NexGen–NexGen
C	MP	F	No	2,561	–	1,358	576	627	Evolution MP–Evolution MP
				546	–	438	282	–	Advance–Advance
C	MP	F	Yes	399	–	117	282	–	Evolution MP–Evolution MP
C	M	F	No	37,811	1,155	16,628	24,028	–	Vanguard Complete–Vanguard Complete
				31,435	2,805	15,364	13,266	–	PFC/Sigma–PFC/Sigma
				21,740	852	14,929	5,959	–	NexGen–NexGen
				18,731	–	7,044	11,687	–	Genesis II–Genesis II
				13,757	–	13,418	–	339	Columbus–Columbus
				11,672	1,787	6,691	2,976	218	Triathlon–Triathlon
				7,517	–	6,953	196	368	Persona–Persona
				5,832	–	3,034	2,798	–	TC–plus–TC–plus
				5,678	–	5,160	518	–	Attune–Attune
				3,875	–	1,198	2,677	–	ACS–ACS
				3,754	–	3,006	748	–	Balansys–Balansys
				2,305	–	2,172	133	–	Innex–Innex
				427	–	280	–	147	Unity–Unity
C	M	F	Yes	21,468	14,000	3,612	3,856	–	PFC/Sigma–PFC/Sigma
				10,704	4,944	643	5,117	–	Vanguard Complete–Vanguard Complete
				5,766	4,207	1,559	–	–	NexGen–NexGen
				5,099	1,905	2,947	247	–	Triathlon–Triathlon
				3,056	–	662	2,394	–	Genesis II–Genesis II
C	P	F	No	55,367	–	15,547	39,820	–	NexGen–NexGen
				37,974	–	2,243	35,731	–	Genesis II–Genesis II
				7,665	–	2,858	4,807	–	Triathlon–Triathlon
				6,648	–	1,816	2,251	2,581	Persona–Persona
				5,845	–	1,823	4,022	–	PFC/Sigma–PFC/Sigma
				3,809	–	993	2,816	–	Balansys–Balansys
				2,726	–	1,904	426	396	Attune–Attune
				601	–	396	205	–	ACS–ACS
				536	–	318	–	218	Columbus–Columbus
C	P	F	Yes	14,523	548	2,004	11,971	–	NexGen–NexGen
				12,204	–	226	11,978	–	Genesis II–Genesis II
				8,729	977	806	6,946	–	PFC/Sigma–PFC/Sigma
				1,980	–	848	1,132	–	Triathlon–Triathlon
				1,462	–	1,279	183	–	Balansys–Balansys
				1,273	–	299	649	325	Attune–Attune
				794	–	149	543	102	Persona–Persona
H	M	F	No	3,134	164	310	2,660	–	Vanguard Complete–Vanguard Complete
				3,074	1,630	580	864	–	NexGen–NexGen
				807	291	516	–	–	PFC/Sigma–PFC/Sigma
				430	160	270	–	–	Triathlon–Triathlon
				478	–	325	–	153	Columbus–Columbus
H	M	F	Yes	4,984	4,851	133	–	–	NexGen–NexGen
				624	159	–	465	–	Vanguard Complete–Vanguard Complete
H	P	F	Yes	333	–	123	210	–	Genesis II–Genesis II
U	M	F	No	2,830	1,091	166	1,573	–	Triathlon–Triathlon

F = Fixation: C = cemented; H = hybrid; U = uncemented.

C = Congruency: F = fully; MP = medial pivot; M = minimally; P = posterior.

M = Mobility: F = fixed.

P = Patella used

### Inclusion of TK implants

Table 2 shows the femoral–tibial brand name combinations for the 47 specific TK implants included in each of the 11 TK constructs, as well as the number of procedures in which these TK implants were used. Most included TK-implant procedures were registered in the LROI (n = 207,017) followed by the EPRD (n = 139,940), the DKR (n = 41,526), and the RIAP (n = 5,471). In 4 TK constructs, only 1 specific TK implant was used, while the TK construct “cemented, minimal congruent, fixed, and no patella usage” included the highest number of specific TK implants, namely 13. 3 TK constructs were used in all 4 registries: (i) the cemented, minimal congruent, fixed, without patella (including 13 different TK implants); (ii) the cemented, posterior stabilized, fixed, with patella (including 7 different TK implants), and (iii) the hybrid, minimal congruent, fixed, without patella (including 5 different TK implants). 5 TK constructs were used in 3 registries, and 3 TK constructs were used in 2 registries.

### Use of UK implants across registries

The percentage of UK implants used was rather similar across registries: highest in the DKR (15.3%), followed by the LROI (13.3%), RIAP (12.4%), and the EPRD (12.1%).

### Comparing patient characteristics for specific TK implants between registries

Overall, patients receiving the 47 specific TK implants were on average 68 years old (SD 7.3 years), with a mean BMI of 30 (SD 3.4), 34% were male, and 81% had the diagnosis osteoarthritis.

Of these 47 TK implants, statistically significant differences in age between registries were found for 29 (62%) TK implants, 30 (63%) had differences in BMI, 23 (49%) in percentages of male sex, and 42 (89%) in percentage of patients with osteoarthritis (Tables 3 and 4). Only 1 TK implant, Genesis II–Genesis II (hybrid/fixed/posterior stabilized/with patella) had no statistically significant difference between registries for any of these patient characteristics.

**Table 3:** Patient characteristics (age and BMI) across registries for the 47 TK implants analyzed in the 11 TK constructs

TK construct and TK implant	n	Mean age				Mean BMI			
		DKR	EPRD	LROI	RIAP	DKR	EPRD	LROI	RIAP
C/F/F/No patella									
NexGen–NexGen	991	–	72	72	–	–	31	29 **^a^**	–
C/MP/F/No patella									
Evolution MP–Evolution MP	2,561	–	68	69	72 **^b^**	–	31	29 **^a^**	–
Advance–Advance	546	–	71	71	–	–	31	–	–
C/MP/F/Patella									
Evolution MP–Evolution MP	399	–	65	68	–	–	30	29	–
C/M/F/No patella									
Vanguard Complete–Vanguard Complete	37,811	68	67 **^b^**	70	–	29	29 **^b^**	31	–
PFC/Sigma–PFC/Sigma	31,435	68	70 **^b^**	69	–	29	31 **^b^**	30	–
NexGen–NexGen	21,740	68	70 **^b^**	68	–	29	31 **^b^**	29	–
Genesis II–Genesis II	18,731	–	68	69 **^a^**	–	–	31	30 **^a^**	–
Columbus–Columbus	13,757	–	69	–	72	–	31	–	–
Triathlon–Triathlon	11,672	67 **^d^**	69	69	72 **^c,d^**	30	31 **^b^**	30	–
Persona–Persona	7,517	–	69	65 **^d^**	71 **^b,d^**	–	31 **^a^**	28	–
TC–plus–TC–plus	5,832	–	71 **^a^**	69	–	–	31	31	–
Attune–Attune	5,678	–	66 **^d^**	72 **^a,d^**	–	–	31	29 **^a^**	–
ACS–ACS	3,875	–	67	66	–	–	32	29 **^a^**	–
Balansys–Balansys	3,754	–	70 **^a^**	68	–	–	31	31	–
Innex–Innex	2,305	–	71	74 **^a^**	–	–	31	–	–
Unity–Unity	427	–	70	–	71	–	31	–	–
C/M/F/Patella									
PFC/Sigma–PFC/Sigma	21,468	70	70	69 **^b^**	–	30	31 **^b^**	30	–
Vanguard Complete–Vanguard Complete	10,704	67 **^b^**	70	68	–	30	31 **^b^**	30	–
NexGen–NexGen	5,766	69 **^a^**	71	–	–	30	31 **^a^**	–	–
Triathlon–Triathlon	5,099	67 **^a^**	68	68	–	30	31	30	–
Genesis II–Genesis II	3,056	–	67	68	–	–	32 **^a^**	30	–
C/P/F/No patella									
NexGen–NexGen	55,367	–	69	69 **^a^**	–	–	31	29 **^a^**	–
Genesis II–Genesis II	37,974	–	69	69	–	–	31	30 **^a^**	–
Triathlon–Triathlon	7,665	–	70	70	–	–	31	30 **^a^**	–
Persona–Persona	6,648	–	70	68 **^a^**	71	–	30	29 **^a^**	–
PFC/Sigma–PFC/Sigma	5,845	–	70	69 **^a^**	–	–	31	30 **^a^**	–
Balansys–Balansys	3,809	–	70	69 **^a^**	–	–	31	29 **^a^**	–
Attune–Attune	2,726	–	69	69	72 **^b^**	–	31	30	–
ACS–ACS	601	–	69	66 **^a^**	–	–	32	31	–
Columbus–Columbus	536	–	68	–	72 **^a^**	–	32	–	–
C/P/F/Patella									
NexGen–NexGen	14,523	68	69 **^a^**	68	–	30	32 **^b^**	29	–
Genesis II–Genesis II	12,204	–	69	67 **^a^**	–	–	30	30	–
PFC/Sigma–PFC/Sigma	8,729	68 **^a^**	69	69	–	30	32 **^b^**	30	–
Triathlon–Triathlon	1,980	–	70	68 **^a^**	–	–	31 **^a^**	30	–
Balansys–Balansys	1,462	–	69	66 **^a^**	–	–	32	29 **^a^**	–
Attune–Attune	1,273	–	69	69	71 **^b^**	–	31	30	–
Persona–Persona	794	–	68	66 **^a^**	70	–	31	29 **^a^**	–
H/M/F/No patella									
Vanguard Complete–Vanguard Complete	3,134	67	68	69	–	30	31	29 **^a^**	–
NexGen–NexGen	3,074	69	68 **^a^**	69	–	29 **^b^**	31	31	–
PFC/Sigma–PFC/Sigma	807	69	68	–	–	29 **^a^**	31	–	–
Triathlon–Triathlon	430	70	69	–	–	30	30	–	–
Columbus–Columbus	478	–	70	–	70	–	31	–	–
H/M/F/Patella									
NexGen–NexGen	4,984	68	68	–	–	30 **^a^**	31	–	–
Vanguard Complete–Vanguard Complete	624	67	–	69	–	30	–	30	–
H/P/F/Patella									
Genesis II–Genesis II	333	–	69	66	–	–	30	31	–
U/M/F/No patella									
Triathlon–Triathlon	2,830	67	66 **^a^**	68	–	30	31	29 **^b^**	–

a Statistically significantly difference from 1 other registry;

b Statistically significantly different from 2 other registries;

c Statistically significantly different from 3 other registries;

d Clinically relevant difference.

**Table 4:** Patient characteristics (% age and osteoarthritis) across registries for the 47 TK implants analyzed in the 11 TK constructs

TK construct and TK implant	n	Proportion male (%)				Proportion osteoarthritis (%)			
		DKR	EPRD	LROI	RIAP	DKR	EPRD	LROI	RIAP
C/F/F/No patella									
NexGen–NexGen	991	–	28	22	–	–	83 **^a,d^**	100 **^d^**	–
C/MP/F/No patella									
Evolution MP–Evolution MP	2,561	–	34	34	29	–	93 **^b^**	100	99
Advance–Advance	546	–	41 **^d^**	10 **^a,d^**	–	–	89 **^a,d^**	100 **^d^**	–
C/MP/F/Patella									
Evolution MP–Evolution MP	399	–	19 **^a,d^**	33 **^d^**	–	–	89 **^a,d^**	100 **^d^**	–
C/M/F/No patella									
Vanguard Complete–Vanguard Complete	37,811	40	48 **^d^**	33 **^b,d^**	–	100 **^d^**	78 **^b,d^**	93 **^d^**	–
PFC/Sigma–PFC/Sigma	31,435	47 **^b,d^**	36 **^d^**	34 **^d^**	–	83 **^d^**	85 **^d^**	100 **^b,d^**	–
NexGen–NexGen	21,740	44	37 **^b^**	43	–	85 **^b,d^**	94	100 **^d^**	–
Genesis II–Genesis II	18,731	–	34	35	–	–	93 **^a^**	100	–
Columbus–Columbus	13,757	–	33	–	32	–	87 **^a,d^**	–	99 **^d^**
Triathlon–Triathlon	11,672	45	38 **^d^**	36 **^d^**	53 **^c,d^**	79 **^c,d^**	82 **^d^**	100 **^d^**	98 **^d^**
Persona–Persona	7,517	–	39 **^d^**	56 **^b,d^**	38 **^d^**	–	93 **^b^**	100	98
TC–plus–TC–plus	5,832	–	37 **^a^**	30	–	–	80 **^a,d^**	100 **^d^**	–
Attune–Attune	5,678	–	41	38	–	–	89 **^a,d^**	100 **^d^**	–
ACS–ACS	3,875	–	29 **^a^**	37	–	–	79 **^a,d^**	100 **^d^**	–
Balansys–Balansys	3,754	–	32	32	–	–	88 **^a,d^**	100 **^d^**	–
Innex–Innex	2,305	–	35	27	–	–	87–	–	
Unity–Unity	427	–	29 **^d^**	–	42 **^d^**	–	79 **^a,d^**	–	91 **^d^**
C/M/F/Patella									
PFC/Sigma–PFC/Sigma	21,468	39 **^b^**	34	30	–	86 **^d^**	86 **^d^**	100 **^b,d^**	–
Vanguard Complete–Vanguard Complete	10,704	41 **^b,d^**	26 **^d^**	30 **^d^**	–	81 **^d^**	91 **^d^**	100 **^d^**	–
NexGen–NexGen	5,766	38	35	–	–	94	85 **^a^**	–	–
Triathlon–Triathlon	5,099	38 **^d^**	40 **^d^**	25 **^b,d^**	–	81 **^d^**	77 **^d^**	100 **^b,d^**	–
Genesis II–Genesis II	3,056	–	30	29	–	–	93	100	–
C/P/F/No patella									
NexGen–NexGen	55,367	–	32 **^a^**	36	–	–	89 **^a,d^**	100 **^d^**	–
Genesis II–Genesis II	37,974	–	35	37	–	–	89 **^a,d^**	100 **^d^**	–
Triathlon–Triathlon	7,665	–	36	36	–	–	79 **^a,d^**	100 **^d^**	–
Persona–Persona	6,648	–	39	41	38	–	78 **^d^**	100 **^d^**	96 **^d^**
PFC/Sigma–PFC/Sigma	5,845	–	33 **^a^**	41	–	–	88 **^a,d^**	100 **^d^**	–
Balansys–Balansys	3,809	–	43	36 **^a^**	–	–	96 **^a^**	100	–
Attune–Attune	2,726	–	38	46 **^b,d^**	32 **^d^**	–	89 **^b,d^**	100 **^d^**	98 **^d^**
ACS–ACS	601	–	36	32	–	–	89 **^d^**	0 **^d^**	–
Columbus–Columbus	536	–	34	–	29	–	87	–	94
C/P/F/Patella									
NexGen–NexGen	14,523	29	30	31	–	77 **^b,d^**	93 **^d^**	100 **^d^**	–
Genesis II–Genesis II	12,204	–	18 **^a,d^**	31 **^d^**	–	–	86 **^a,d^**	100 **^d^**	–
PFC/Sigma–PFC/Sigma	8,729	40 **^b^**	32	35	–	77 **^d^**	74 **^d^**	100 **^b,d^**	–
Triathlon–Triathlon	1,980	–	34	34	–	–	72 **^a,d^**	100 **^d^**	–
Balansys–Balansys	1,462	–	37 **^d^**	21 **^a,d^**	–	–	99	100	–
Attune–Attune	1,273	–	39	39	33	–	57 **^b,d^**	100 **^d^**	98 **^d^**
Persona–Persona	794	–	39 **^d^**	28 **^d^**	29 **^d^**	–	91 **^b^**	100	98
H/M/F/No patella									
Vanguard Complete–Vanguard Complete	3,134	40	46	38	–	87 **^d^**	92	100 **^b,d^**	–
NexGen–NexGen	3,074	42 **^d^**	50 **^d^**	29 **^b,d^**	–	92 **^d^**	82 **^b,d^**	100 **^d^**	–
PFC/Sigma–PFC/Sigma	807	43	41	–	–	86	86	–	–
Triathlon–Triathlon	430	49 **^d^**	38 **^d^**	–	–	86 **^d^**	70 **^a,d^**	–	–
Columbus–Columbus	478	–	34	–	34	–	92 **^a^**	–	100
H/M/F/Patella									
NexGen–NexGen	4,984	41	39	–	–	90 **^d^**	74 **^a,d^**	–	–
Vanguard Complete–Vanguard Complete	624	40 **^d^**	–	25 **^a,d^**	–	74 **^a,d^**	–	100 **^d^**	–
H/P/F/Patella used									
Genesis II–Genesis II	333	–	44	48	–	–	98	100	–
U/M/F/No patella									
Triathlon–Triathlon	2,830	43	47 **^d^**	37 **^a,d^**	–	87 **^d^**	93	100 **^b,d^**	–

a Statistically significantly difference from 1 other registry;

b Statistically significantly different from 2 other registries;

c Statistically significantly different from 3 other registries;

d Clinically relevant difference.

As for clinically relevant differences in patient characteristics when the same TK implant was used, age was different in 3 of the 47 (6%) TK implants, percentage of male sex in 17 (36%), percentage of patients with diagnosis of osteoarthritis in 34 (72%), whilst no differences in BMI were found.

## Discussion

This is the first multi-registry study to compare the use of specific TK implants across registries and by comparing their use in comparable patients characterized by age, sex, BMI, and diagnosis of osteoarthritis. This is essential for comparison of safety and performance of the implant between registries. Only 53 (7%) of the 813 specific TK implants were used in a single registry, suggesting that pooling data across registries to detect any safety concerns is possible for most (93%) TK implants. Of the 47 TK implants used ≥ 100 times in ≥ 2 registries, statistically significant differences in patient characteristics were found in 62% of the TK implants for age, 77% for BMI, 49% for male sex, and 89% for diagnosis of osteoarthritis. Only a small number of these statistically significant differences in age and male sex were deemed clinically relevant, none for BMI, but a large proportion (72%) of the differences in osteoarthritis diagnosis. These findings suggest that when comparing the performance for specific TK implants across registries potential differences in patient characteristics should be considered, particularly regarding diagnosis.

Most studies investigating differences in patient characteristics across registries did not consider specific TK implants but analyzed all TK implants combined [11,12], and found considerable differences between countries in preoperative patient characteristics (e.g., age and BMI) and pain levels. Our study contributes to this literature by providing a more comprehensive analysis of differences in patient characteristics for specific TK implants. For clinicians, such detailed analysis on the TK-implant level will likely be more clinically relevant in guiding implant selection, as clinicians select implants based on their performance but need these patient characteristics to put the performance in context. For example, if the revision risk of a specific implant is good but based on a relatively older population while the patient concerned is much younger, then it is uncertain whether the implant will perform similarly in that patient. Such TK-implant-level information is also relevant for regulators to better interpret the safety and performances of TK implants on the market across registries [20], as elderly patients, for example, may have lower remaining life expectancy, and surgeons may be less likely to revise given the higher risks associated with surgery [21].

Even though we found statistically significant differences in age, BMI, percentage of males, and osteoarthritis diagnosis across registries for many TK implants, the question is whether these differences are also clinically relevant. In a large study population, even very small differences can be detected as statistically significant, though they may not be clinically relevant [22]. As clinical differences are more relevant for clinicians, we also determined the clinically relevant differences by applying commonly used thresholds [18,19]. Only a small number of TK implants showed clinically relevant differences in age, BMI, and male sex, but differences in osteoarthritis diagnosis remained for a large proportion of TK implants. This suggests that most TK implants are used in similar patient groups except for diagnosis. This is in line with research showing differences in treatment approaches for knee osteoarthritis between countries, influenced by several factors such as variations in healthcare systems, guidelines and preferred approaches, economic factors, and cultural preferences [23,24]. The relatively high differences in osteoarthritis diagnosis might be caused by differences in definitions or the classification used. While the DKR, EPRD, and RIAP included primary osteoarthritis to calculate the percentage, the LROI included both primary and secondary osteoarthritis. Even though we tried to harmonize as much as possible across registries, these differences reflect the heterogeneity in definitions and methods across registries and show the need for further harmonization for better comparison.

To allow for early detection of safety issues in specific implants, it is often recommended that data across registries should be pooled to increase the number of implants at risk for statistical analysis and thereby statistical power [2,25]. Another advantage of pooling data across registries is that it might better represent real-world performance of this specific device across all patients in which it is used. On the other hand, if we want to know the revision risk for a specific implant in a specific patient population, we would need to include only specific patients to arrive at the best revision risk estimate, akin to what we do when pooling data in a meta-analysis. In addition, pooling of data is complicated by large heterogeneity in methods used across registries, definitions, and outcomes collected, which negatively impacts the ability to pool data [2]. The current study shows that if harmonization across registries in methods and collected outcomes can be reached, pooling of data will be possible for the majority of TK implants (93%), as only 7% of TK implants were used in a single registry, and this is particularly valuable for TK implants with limited sample size.

Combining data from multiple registries may also increase the heterogeneity of the included data due to factors other than recorded patient characteristics, where using data from a single registry may limit this heterogeneity, which makes interpretation more straightforward. For instance, revision tendencies can vary between countries, which influences the estimated performance (i.e., revision risks) of specific TK implants. When using data from an individual registry, such differences in tendencies to revise may be smaller, although between-hospital variations in revision thresholds may still exist as well as differences in operative volumes of individual surgeons and hospitals, all known as factors influencing revision risks [26,27]. Although data pooling has its limitations, we believe that pooling data should be recommended, to increase the number of implants for statistical power and thus to better represent real-world performance of a specific implant.

### Limitations

First, we were limited in the patient characteristics that could be compared between registries, where more factors (e.g., American Society of Anesthesiologists [ASA] classification) may affect the safety and performance of primary TK implants, and are therefore important to take into account when comparing the performance of TK implants [28]. Second, the frequency of UK implants used in a registry may affect differences found in patient characteristics where it is known that UK implants are more commonly used, for instance in younger patients [29]. However, as the variation in UK implants used across registries was relatively small, the impact is likely negligible. Third, there could have been selection bias because not all TK implants used in patients were reported in registries (i.e., TK-implant-level completeness ranges from 58.7% to 97%). Lastly, we limited our analysis to 4 national registries where a larger number of regional, national, and multi-country registries exist [2]. Including additional registries could have resulted in a higher number of specific TK implants used across multiple registries for which patient characteristics could be compared.

### Conclusion

Most TK implants were used in multiple registries, indicating that if harmonization of data collection across registries is achieved, this will enable pooling of data across registries for detection of safety concerns, particularly for those TK implants with limited sample size within a registry. In addition, differences in characteristics of patients receiving the same TK implant across registries were found, which should be considered when comparing the performance of the same TK implant across registries and may assist clinicians in implant selection for specific patients.

## Notes

LAH: conceptualization, data curation, formal analysis, methodology, project administration, writing—original draft. RGHHN: conceptualization, funding acquisition, writing—review and editing. PJM-vdM: conceptualization, funding acquisition, data curation, methodology, project administration, writing—review and editing. ABP, AWG, EBK, MLL, EC, LvS, MT, RV, YW: data curation and writing—review and editing.

The authors would like to thank Paola Laricchiuta and Filippo Boniforti for their contribution to the Italian Arthroplasty Registry and the Danish, Dutch, German, and Italian arthroplasty registries for providing the data, as well as all the operating theatre staff at participating hospitals throughout these countries for collecting the data.

Handling co-editors: Marianne Westbergand Robin Christensen

*Acta* thanks Oystein Johannes Gothesen and Peter Lawrence Lewis for help with peer review of this manuscript.
